# Prior-Knowledge-Guided Graph Attention Network for Fault Diagnosis of Engine Valve Clearance

**DOI:** 10.3390/s26113565

**Published:** 2026-06-03

**Authors:** Mingyu Li, Jingqian Wen, Xiaonan Yang, Yaoguang Hu, Xinlong Li, Zhongjie Shi

**Affiliations:** 1 Laboratory of Industrial and Intelligent Systems Engineering, School of Mechanical Engineering, Beijing Institute of Technology, Beijing 100081, China; 2 Testing Service Center of Xishan Campus, Experimental Center of Vehicles, Beijing Institute of Technology, Beijing 100081, China

**Keywords:** graph neural network, attention mechanism, prior knowledge, engine valve clearance, small sample, fault diagnosis

## Abstract

Fault diagnosis of diesel engines is a critical task in the operation and maintenance of complex equipment. Diesel engine fault diagnosis technology based on deep learning has seen widespread development due to its powerful feature learning and fault classification capabilities. However, traditional data-driven deep learning models cannot explicitly uncover relationships between signals, which hinders better fault information capture. Therefore, this paper proposes a diesel-engine valve-clearance fault diagnosis method driven by a combination of knowledge and data. Firstly, the original signals are converted into graph data with a topological structure based on the spatiotemporal relationships of events occurring within the cylinder, thereby uncovering the intrinsic structural information of the samples. Then, the graph structure is input into a graph convolutional attention network to extract features and learn fault patterns. Valve fault experiments were conducted on a diesel engine test bench, and the results indicate that the proposed knowledge and data-driven deep learning fault diagnosis model achieves better diagnostic performance and clearer interpretability compared to traditional data-driven deep learning fault diagnosis models, and it still has a relatively high accuracy in a diagnostic environment with scarce data.

## 1. Introduction

As a widely used power equipment, diesel engines play an important role in fields such as shipping and military industry [1]. Due to their complex structure and harsh operating environments, they have a relatively high probability of failure. During the operation of a diesel engine, the valves are subject to repeated impacts due to the reciprocating power process of the cylinder. Over time, valve springs may gradually deteriorate and deform, causing wear and carbon buildup on the valves, which leads to abnormal increases in valve clearance. This reduces the efficiency of cylinder flow control and affects the normal energy conversion process of the diesel engine, causing a decline in engine power [2,3,4,5]. Furthermore, the continuous abnormal increase in valve clearance may result in severe faults such as cylinder impact or valve breakage, causing significant economic losses and even threatening personal safety [6]. Therefore, it is essential to study diagnostic methods for abnormal valve clearance in diesel engines.

Currently, deep learning (DL) algorithms have seen exponential growth in their use for mechanical fault diagnosis research over the past decade due to their high accuracy, efficient data representation, and automatic feature extraction and selection [7,8,9]. Unlike traditional machine learning, DL algorithms stack multiple layers to express complex objective functions, uncover intrinsic relationships between variables, and improve model generalization performance [10]. DL algorithms, represented by various neural networks, have powerful feature extraction capabilities, allowing for automatic representation learning from large datasets and strong adaptability [11]. Deep Belief Networks (DBN), one of the classic DL models [12,13], have been applied early on in fault diagnosis [14,15]. DBN can extract effective fault features from feature sets constructed for specific tasks. Unlike traditional shallow machine learning algorithms, DBN can learn high-level feature representations from large amounts of data [16,17]. By directly utilizing raw signals or spectra, end-to-end intelligent diagnostic models can be established, reducing reliance on expert experience and knowledge. Early studies such as Deep Belief Networks (DBN) were capable of automatically extracting features. However, their black-box nature and the limitation of being unable to explicitly model the relationship between signals led to the exploration of structured modeling methods like graph neural networks.

Deep learning (DNN) has achieved significant success in mechanical fault diagnosis, but there are still associated challenges [18,19,20]. It is widely believed that deep learning (DNN) can learn correlations between inputs. However, this self-learning process of feature representation cannot explicitly capture the relationships between signals [21]. In mechanical fault diagnosis, the relationships between monitoring signals change significantly with the health state of the machine. Therefore, modeling and learning these signal relationships will be effective for machine fault diagnosis [22].

While these DNN-based methods effectively capture hidden features of conventional data (e.g., images, time series), most methods overlook the interdependencies between data from multiple sensors or various physical measurements [23,24]. In recent years, graph neural networks (GNN) have gained significant attention from researchers due to their ability to establish associations between data [25]. By aggregating information from node neighbors at any depth, GNN can more effectively extract and infer data relationships [26]. For instance, Li et al. used Horizontal Visibility Graph (HVG) and GNN to propose a new bearing fault diagnosis model. The HVG algorithm converts time series samples into graphs with specific conditional topologies, providing additional valuable information for classification compared to pure numerical data, demonstrating that GNN models outperform RNN models in bearing fault diagnosis [27]. Graph Convolutional Networks (GCN) [28,29,30], a variant of GNN, use associative graphs to establish data associations, speeding up training and improving model performance. For instance, Zhou [31]. proposed a GCN-based fault diagnosis method for rotating machinery using multi-sensor data. To diagnose wind turbine gearbox issues, Yu [32] proposed a Fast Deep Graph Convolutional Network (FDGCN), which efficiently and adaptively learns discriminative fault features from initial graph inputs, and then uses these learned features to classify relevant fault types. Zhang [33] applied Deep Convolutional Graph Neural Networks (DGCN) to diagnose acoustic faults of roller bearings. However, most existing GNN-based fault diagnosis methods construct graphs mainly from data-driven relationships, such as sample similarity, sensor correlation, visibility rules, or generic graph construction strategies including KNNGraph, RadiusGraph, and PathGraph [34,35]. Xiao et al. proposed a GNN-based bearing fault detection method in which graph nodes and edges were constructed according to sample similarity, demonstrating the effectiveness of similarity-based graph modeling for bearing fault diagnosis [35]. Li et al. systematically reviewed GNN-based fault diagnosis methods and pointed out that most existing graph construction strategies rely on data similarity, sensor relationships, or predefined generic graph structures rather than explicit physical event mechanisms [36]. These methods have shown effectiveness in representing non-Euclidean relationships in mechanical signals. Nevertheless, such graph structures are usually determined by statistical relationships among samples or sensors. Under small-sample conditions, similarity estimation may be unstable, and the obtained graph topology may not always correspond to the actual physical mechanism of the monitored system.

To improve the physical interpretability of graph models, several recent studies have attempted to incorporate prior knowledge into GNN-based diagnosis or anomaly detection. Deng et al. proposed a physics-informed gated recurrent graph attention unit network for anomaly detection in industrial cyber-physical systems, where physical relations were introduced through graph regularization to guide graph structure learning [37]. Liu et al. developed a semi-heterogeneous graph-perception network for industrial fault recognition and root-cause diagnosis, in which industrial topology information was used to support graph-based fault interpretation [38]. Tao et al. proposed a knowledge-and-data-fusion method for associated fault diagnosis of power supply systems, where prior fault knowledge was used to construct fault association graphs and assist graph matching [39]. These studies indicate that incorporating domain knowledge into graph modeling is an important direction for improving interpretability and robustness. However, most of these methods embed prior knowledge through regularization constraints, sensor-level spatial topology, process flow relationships, or fault association graphs. They do not directly encode the ordered causal-temporal sequence of the working cycle into the graph topology.

To address the above issues, this paper proposes a prior-knowledge-guided Graph Attention Network (GAT) method for valve-clearance fault diagnosis. In the proposed method, the cylinder-head vibration signal is first synchronized in the crank-angle domain. Key event-centered vibration segments within one working cycle are then extracted as graph nodes, and directed edges are constructed according to the physical occurrence sequence of these events. In this way, the working-cycle event process is embedded into the graph topology. The constructed event-driven graph reduces the dependence on data-driven similarity estimation under small-sample conditions, preserves the physical correspondence between vibration segments and valve-related events, and provides a basis for interpreting the learned attention weights. The main contributions of this work are summarized as follows:

- A prior-knowledge-guided directed graph is constructed by treating event-centered vibration windows as graph nodes and connecting them according to the physical occurrence sequence of the working cycle. This design preserves the physical correspondence between vibration segments and valve-related events.
- A GAT-based diagnostic model is developed to learn discriminative graph-level representations from the constructed event-driven graph samples. The learned attention weights are further visualized, providing an interpretable basis for analyzing event-to-event relationships related to valve-clearance faults.
- Fault simulation experiments are conducted on an eight-cylinder diesel engine test bench under full-load conditions. The results verify that the proposed event-driven graph modeling strategy achieves effective small-sample diagnostic performance and provides clearer physical interpretability.

## 2. Related Work

### 2.1. Mathematical Notation of Graph

The graph is a kind of data structure which is used to describe a specific relationship between things [40]. A graph is composed of vertices (also called nodes) and edges between vertices; therefore, graph G can be expressed as a two-tuple composed of point set vertices $\left(V\right)$ and edge set edges $\left(E\right),$ denoted by $G\left(V,E\right)$ in which: $V=v_{1},v_{2},v_{3},\cdots v_{n}$ where $v_{i}$ is vertex i. The graph usually has node features $H\;\in \;R_{n}\times d$, where n is the number of vertices and d denotes the dimension of vertices eigenvector; $e_{ij}\in \;E$ denotes an edge from vertices $v_{i}$ to $v_{j}$; if the connection order between the vertices is not considered, the edge ${e\;}_{ij\;}$ is said to be an undirected edge, that is, $v_{i}$ and $v_{j\;}$ are adjacent to each other. The adjacency matrix *A* of the graph G is represented in Equation (1):

(1) $$A_{ij}=\left\{\begin{array}{l} 1,\;if\;e_{ij}\in E\\ 0,\;if\;e_{ij}\notin E\; \end{array}\right.$$

where $A_{ij}=1$ indicates that there is an edge from node $v_{i}$ to node $v_{j}$, and $A_{ij}=0$ otherwise.

### 2.2. Graph Neural Networks

Graph neural network (GNN) is a type of neural network model specifically designed to process graph-structured data [41]. Traditional neural networks such as Convolutional Neural Network (CNN) and Recurrent Neural Network (RNN) require the features of nodes to be arranged in sequence, which makes them ineffective for handling graph-structured inputs. In contrast, GNNs are a type of model in which the dependencies in the graph are captured through the message passing between nodes within the network. GNNs update the hidden state of nodes by aggregating and weighting the features of neighboring nodes. In a graph structure, the state vector of a node is defined by its own features and the features of its connected nodes [42]. GNNs aim to learn the embedding $h_{v}\in R^{s}$, which encapsulates information from the node itself and its neighbors. The vertex state vector $h_{v}$ can be used to generate an output $o_{v}$, which is typically the label $v_{0}$ of the vertex. The local transformation function $f\left(\cdot \right)$ updates the vertices’ state based on the inputs from neighbors, while the local output function $g\left(\cdot \right)$ describes the pattern of output generation. $h_{v}$ and $o_{v}$ are generated according to the following equations:

(2) $$h_{v}=f(x_{v},x_{co|v|},h_{ne|v|},x_{ne|v|})$$

(3) $$o_{v}=g(h_{v},x_{v})$$

where $x_{v}$ is the feature vector of node v, $x_{co\left|v\right|}$ denotes the edge features connected to node v, $h_{ne\left|v\right|}$ denotes the hidden states of neighboring nodes, and $x_{ne\left|v\right|}$ denotes the feature vectors of neighboring nodes.

If all eigenvectors, all eigenvectors of edges, all state vector of neighbors and all eigenvectors of neighbors are superimposed together, they are represented by matrices X, $X_{co}$, $H_{ne}$ and $X_{ne}.$ Then, a more compact representation can be obtained:

(4) $$H=F(X,X_{co},H_{ne},X_{ne})$$

(5) O=G(H,X)

where $F\left(\cdot \right)$ represents the global transformation function and $G\left(\cdot \right)$ represents the global output function, which are the superposition of $f\left(\cdot \right)$ and $g\left(\cdot \right)$, respectively. Based on Banach theorem, a GNN adopts the following iterative formula to calculate vertices’ states

(6) $$H^{t+1}=F(H^{t},X)$$

where $H^{t}$ represents the state of the t th period, and the system iterated according to the above equation converges to the final, fixed point solution.

### 2.3. Graph Attention Network (GAT)

The attention mechanism has become a highly popular concept in neural networks in recent years [43]. It offers advantages such as fewer parameters, faster processing, and improved performance, and has been widely applied in various fields, including machine translation and image recognition [44,45]. Without prior knowledge, the attention mechanism enables models to autonomously learn the differences in importance between input information. Guided by attention coefficients, the model focuses more on the important parts of the input while ignoring less significant information, leading to more efficient feature learning. Similarly, the attention mechanism can be applied to graph-structured data, allowing different weights to be learned for different nodes within a neighborhood, thus enhancing the learning process and improving model performance. Inspired by these studies, the attention mechanism has also been introduced into graph neural networks, such as the Graph Attention Network (GAT).

GAT introduces the attention mechanism into the graph convolution operation [46]. For a graph with a set of node features $h=\left\{{\overset{⃑}{h}}_{1},{\overset{⃑}{h}}_{2}{,\ldots ,\;\overset{⃑}{h}}_{n}\right\},{\overset{⃑}{h}}_{i}\;\epsilon \;R^{d}$, graph attention convolution (GATConv) output is a set of new node characteristics of layer, $h^{\prime }=\left\{{\overset{⃑}{h^{\prime }}}_{1},{\overset{⃑}{h^{\prime }}}_{2}{,\ldots ,\;\overset{⃑}{h^{\prime }}}_{n}\right\},\;{\overset{⃑}{h^{\prime }}}_{i}\epsilon R^{d}$; therefore, the attention coefficient of each node can be represented as:

(7) αij=exp(LeakyReLU(α→T·[θh→i||θh→j]))∑μ∈Niexp(LeakyReLU(α→T·[θh→i||θh→j]))

In the formula, ${\overset{⃑}{\alpha }}^{T}$ represents the parameters of the single-layer feedforward neural network, $\mu \epsilon N_{i}$ represents the neighbors of node i, and $\alpha_{ij}$ represents the attention coefficient between node i and node j. ∥ indicates a linking operation. *θ =* {*W*, a} denotes the trainable parameters of the GAT layer, *W* is the feature transformation matrix, and a is the attention vector used to calculate the attention coefficient. $LeakyReLU\left(\cdot \right)$ is a nonlinear activation function.

According to the calculated attention coefficient, the convolutional layer of the attention map is defined as:

(8) $${{\vec{h}}^{\prime }}_{i}=\sigma (\sum_{\mu \in N_{i}}\alpha_{ij}\theta \;{\vec{h}}_{j})$$

In the formula, σ(·) is the activation function, and $\alpha_{ij}$ is the standardized attention coefficient calculated by the attention mechanism. ${\vec{h^{\prime }}}_{i}$ represents the final learned node representation.

**Figure 1:**
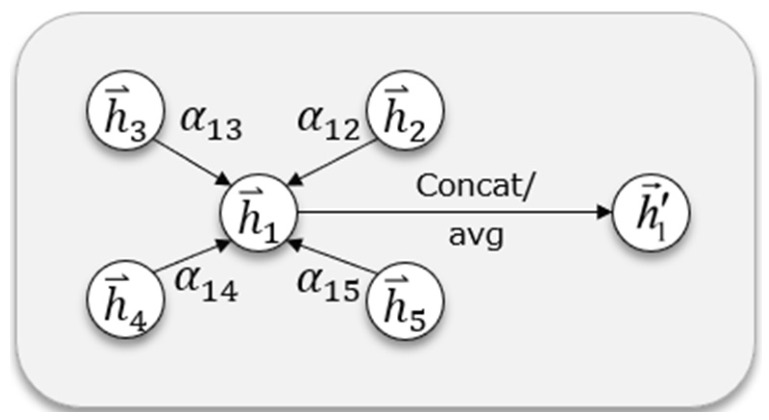
Schematic of the attention mechanism.

The specific process of the GAT layer is shown in Figure 1. In the GAT layer, the original features of node *v* and its neighborhood features are first input into the softmax layer to obtain the attention coefficient of each node. Then, the inner product is performed between the generated attention coefficient and the features of its corresponding neighborhood nodes to obtain the new node feature node v.

## 3. The Proposed Diesel Fault Diagnosis Method Based on a GAT

### 3.1. Overview

**Figure 2:**
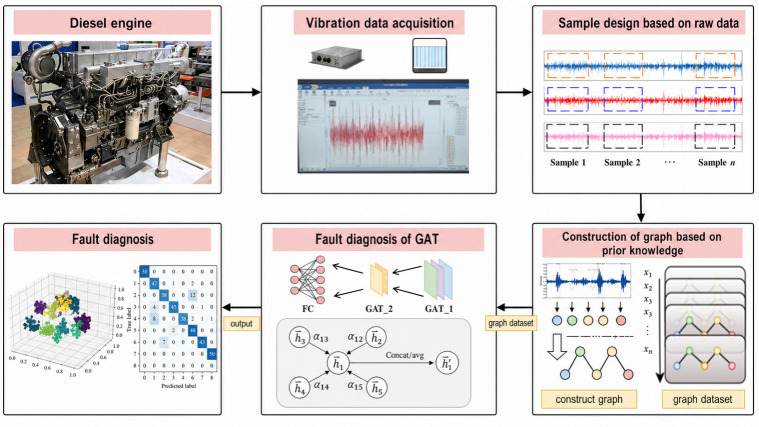
Overview of the proposed method.

The proposed framework for diesel engine valve-clearance fault diagnosis, driven by data and knowledge fusion based on GAT, is illustrated in Figure 2. It mainly comprises two stages: graph construction based on prior knowledge and fault diagnosis based on GAT. Firstly, according to the working mechanism of the diesel engine cylinder, the original vibration signal is segmented according to the event occurrence intervals, converting the Euclidean structure data (original vibration time-domain signal) into non-Euclidean structure data (graph) that contains the prior knowledge of the diesel engine cylinder. In the second stage, the constructed graph is used as the input to the GAT model to diagnose the health status of the diesel engine valve clearance.

### 3.2. Constructing Affinity Graphs from Time Series Based on Prior Knowledge

#### 3.2.1. Analysis of Diesel Engine Vibration Signal Characteristics

The main measurement points of the diesel engine are arranged at the gearbox, crankcase, and cylinder head [47]. More precisely, the measurement point for the gearbox is located on the casing of the gear system, primarily monitoring gear meshing-related frequencies. However, due to buffering from gaskets and other factors, the monitored signal characteristics may be relatively weak. The measurement point for the crankcase is primarily arranged on the same horizontal plane as the crankshaft center. The moving components directly contacting the crankcase include the crankshaft and pistons, so this measurement point mainly monitors the overall vibration of the unit. The cylinder head directly contacts the cylinder of the diesel engine. Since this unit uses a split-type cylinder head, the cylinder head measurement points include the operational status of each cylinder, capable of representing the operating conditions of internal components such as valves, connecting rod-piston assemblies, and ignition conditions [48,49]. Therefore, this study uses cylinder head signals for fault diagnosis and analyzes the vibration characteristics of the cylinder head signals [50].

To capture the impact-related vibration induced by valve events, a triaxial piezoelectric accelerometer (model 1A313E, Donghua Test) was mounted on the cylinder head close to the monitored cylinder. The sensor has a measurement range of ±100 g and a frequency response of 0.5–7000 Hz (±10%). The calibrated sensitivities at 160 Hz are approximately 4.0 mV/(m/s^2^) on all three axes. The sensor is powered by a constant-current source of 2–20 mA (18–30 VDC), with a DC bias voltage of 8–12 V, and is installed through an M5 threaded mounting hole to ensure reliable mechanical coupling. The collected continuous vibration signal was subsequently segmented according to the event-driven strategy described in this study.

The event-window length used for extracting each node feature was selected as approximately 20 crank-angle degrees. At the operating speed of 1500 rpm, a 20° crank-angle window corresponds to approximately 2.22 ms, and this window was extracted around each key event to construct the graph nodes. The selection of this window length was based on the crank-angle-synchronized positions of the key valve-related events and the local transient characteristics of the cylinder-head vibration signal. Previous studies have shown that diesel-engine cycle events, such as combustion, fuel injection, and valve opening/closing, can be identified in the crank-angle domain from surface vibration or acoustic emission signals [51,52]. In addition, valve opening and closing impacts are local transient responses, and their occurrence phases are sensitive to valve-clearance variation. Jiang et al. reported that the commencement phase of valve closing impact advanced by approximately 8°CA to 10°CA under different valve-clearance conditions [53]. Therefore, an approximately 20°CA window was adopted in this study to cover the main event-related vibration response and possible phase fluctuation while avoiding overlap with neighboring event windows. This setting helps each graph node maintain a clear physical correspondence with a specific valve-related event. It should be noted that the 20°CA window was not intended to represent a universal physical duration of each valve event, but was selected as a physically informed empirical window for event-centered feature extraction under the present test condition.

To achieve the node division and structural construction of the graph, this study analyzed the valve phase data of diesel engine cylinder block A1, as shown in Table 1. Taking a complete working cycle as a reference, the sequence of key events of this cylinder is as follows:

Based on the phase sequence of the above-mentioned cylinder actions, a complete working cycle can be divided into five main physical events [54]:

1. Exhaust valve opening: Towards the end of the power stroke, the exhaust valve opens in advance, allowing the high-temperature and high-pressure exhaust gas after combustion to start being discharged, providing favorable initial conditions for the subsequent exhaust stroke, while reducing the pressure inside the cylinder and alleviating the upward resistance of the piston.
2. Intake valve opening: When the exhaust stroke is not yet completed, the intake valve opens in advance, forming a valve overlap area with the exhaust valve. This helps to utilize the negative pressure generated by the exhaust gas flowing out to draw fresh air into the cylinder, thereby enhancing the ventilation efficiency.
3. Exhaust valve closure: After the intake stroke begins, the exhaust valve closes with a delay, further enhancing the complete discharge of exhaust gases and, in conjunction with the intake process, creating a more efficient air replacement effect, thereby improving the intake quality.
4. Intake valve closure: The intake valve closes when the intake stroke is completed and the compression stroke is about to begin. This moment is slightly later than the bottom dead center. The delayed closure can increase the air intake volume in the cylinder by taking advantage of the intake inertia, thereby improving the compression efficiency and combustion effect.
5. Ignition: Before the end of the compression stroke, the fuel self-ignites through high-pressure fuel injection, generating high-temperature and high-pressure gas that pushes the piston to do work. This is a key node for energy conversion in the entire four-stroke cycle, marking the beginning of the power stroke.

#### 3.2.2. The Construction of Graph Based on Prior Knowledge

Based on the analysis of the vibration signal characteristics in the previous section, it can be observed that the signal in the event intervals corresponding to the fault condition undergoes certain changes. Therefore, if we divide the signal according to events and treat each event interval as a node, with nodes connected according to the event occurrence process, this graph data will incorporate some prior mechanistic knowledge of the diesel engine. This approach facilitates accelerating the model training and achieving fault diagnosis based on small sample [55,56,57].

First, we sample the original signal over a working cycle period, dividing it into numerous fixed-length samples. For each sample, we then segment it according to the events of ignition, exhaust valve opening, intake valve opening, exhaust valve closing, and intake valve closing. For each cycle-based sample X, five event-centered signal segments are extracted according to the key physical events, as shown in the following equation:

(9) $$X=\left[\begin{array}{cccc} x_{1}^{1} & x_{2}^{1} & \ldots & x_{m}^{1}\\ x_{1}^{2} & x_{2}^{2} & \ldots & x_{m}^{2}\\ \ldots & \ldots & \ldots & \ldots \\ x_{1}^{i} & x_{2}^{i} & \ldots & x_{m}^{i} \end{array}\right]$$

Here, *x* represents the original sampling points, m is the sample length, and *i* is the event index, $x_{i}$ denotes the signal segment corresponding to the *i*-th physical event, and *i* = 1, 2, …, 5 represents ignition, exhaust valve opening, intake valve opening, exhaust valve closing, and intake valve closing, respectively. Next, min-max normalization is used to preprocess the original signals from different events separately.

(10) $$x_{i}^{nol}=\frac{x_{i}-x_{\min}}{x_{\max}-x_{\min}},i=1,2,\ldots ,m$$

Here, $x_{max}$ and $x_{min}$ are the maximum and minimum values of the corresponding signal segment, $x_{i}^{\mathrm{nol}}$ is the normalized value, and m is the sample length.

**Figure 3:**
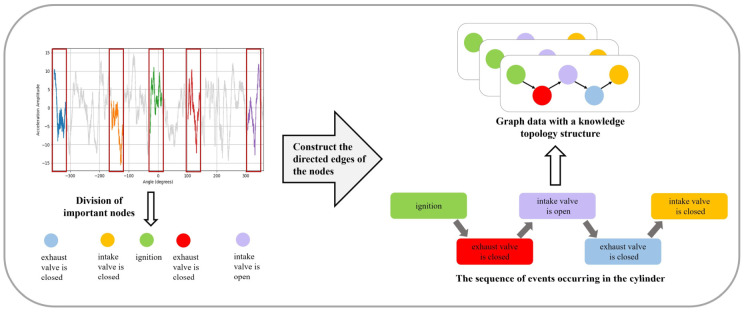
Schematic diagram for the construction of graphs based on prior knowledge.

Next, in this study, each sliding window signal segment is regarded as a node in the graph, and the signals contained in the nodes retain the essential characteristics of the event. Then, in the sequence of events occurring within the cylinder, the directed edges between the nodes are constructed: ignition → exhaust valve opening → intake valve opening → exhaust valve closing → intake valve closing. In this way, the independent samples of the Euclidean space are constructed as a graph with a knowledge topology structure. The graph can be defined as $G\left(V,E\right)$, where $V=\left\{v_{1},v_{2},v_{3}\ldots v_{n}\right\}$ represents a set of vertices, and E represents a set of edges connecting the vertices.The process of constructing the prior-knowledge-guided graph is shown in Figure 3.

### 3.3. Fault Diagnosis Based on the GAT

**Figure 4:**
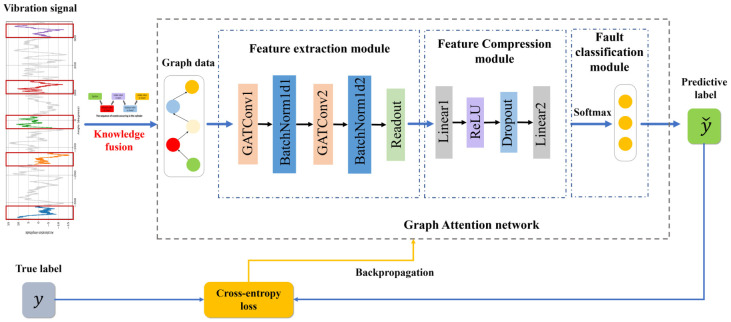
Overall architecture of the model.

To achieve efficient identification of abnormal valve clearance states in diesel engines, this paper designs and constructs a structured fault diagnosis model based on Graph Attention Network (GAT). This model introduces the mechanism knowledge of the intake and exhaust system to construct a graph structure, models the dependency relationship between vibration signal fragments in a topology-aware manner, and strengthens the feature aggregation ability of key nodes through the attention mechanism, thereby achieving more robust classification performance under the condition of small sample. The overall architecture of the model is shown in Figure 4, which integrates the feature extraction module, the feature compression module and the fault classification module.

(1) Feature extraction module

In the feature extraction stage, the model introduces a two-layer graph attention convolution structure (GATConv) to mine the structural associations between nodes layer by layer. The first-layer GAT receives the initial features of the nodes and adaptively allocates the weight coefficients of adjacent nodes through the attention mechanism to achieve effective encoding of local topological relationships. The nonlinear modeling ability is enhanced by integrating the batch normalization (BatchNorm) and the Leaky ReLU activation function. The second layer of GAT further extracts the high-order structure information on the basis of the previous layer. Meanwhile, it enhances the expression and transmission ability of the deep features of the model through the residual connection mechanism to alleviate the problem of vanishing gradient. After the node-level features are encoded through graph convolution, the global mean pooling operation is adopted to compress the graph representation vectors of all nodes into a unified graph level feature vector, achieving the structural representation of the entire signal sample. This graph-level feature, as the core input for fault discrimination, is passed to the subsequent fully connected layer for classification decision-making.

Calculate the attention coefficient $\alpha_{ij}$ of node i and its neighbor node j for each layer of GAT using Formula (7).

The model uses a total of two GAT layers, and its output is:

(11) $$H^{\left(1\right)}=GAT_{\;\;1}(H^{\left(0\right)},A),H^{\left(2\right)}=GAT_{\;\;2}(H^{\left(1\right)},A)$$

Finally, all node features are aggregated into a graph-level representation vector through the average pooling method:

(12) $$h_{graph}=\frac{1}{N}\sum_{i=1}^{N}h_{i}^{\left(2\right)}$$

In the formula, $h_{i}^{\left(2\right)}$ denotes the representation of node *i* after the second GAT layer, *N* is the number of graph nodes, and $h_{graph}$ is the graph-level representation obtained by global mean pooling.

(2) Feature Compression module

In the feature compression stage, the graph-level representation first inputs a two-layer perceptron (MLP) module for processing: The first layer of linear mapping compresses the node features to a low dimension and is activated by ReLU. The second layer of linear mapping maps the feature dimensions to the number of categories and introduces dropout for feature perturbation and regularization.

(13) z1=ReLU(W1hgraph+b1), z1′=Dropout(z1)

(14) $$z_{2}=W_{2}z_{1}^{\prime }+b_{2}$$

where $z_{1}$ and $z_{2}$ denote the outputs of the first and second fully connected layers, respectively, $W_{1}$, $W_{2}$, $b_{1}$ and ${\;b}_{2}$ are learnable parameters.

(3) Fault Classification module

In the fault classification stage, the graph representation vector is sent to the softmax layer to output the predicted probability, achieving the classification and judgment of the valve clearance state, including three states: “normal clearance”, “abnormal intake valve clearance”, and “abnormal exhaust valve clearance”. During the training process, the cross-entropy loss function is adopted as the optimization objective, and the parameters are updated in combination with the Adam optimizer

(15) $$\overset{ˇ}{y}=\mathrm{softmax}\left(z_{2}\right),\;{\overset{ˇ}{y}}_{c}=\frac{\exp(z_{2},c)}{\sum_{k=1}^{C}\exp(z_{2},k)}$$

In the formula, ${\overset{ˇ}{y}}_{c}$ represents the predicted probability that the sample belongs to category c and C denotes the number of classes.

## 4. Experimental Validation

### 4.1. Experimental Setup

**Figure 5:**
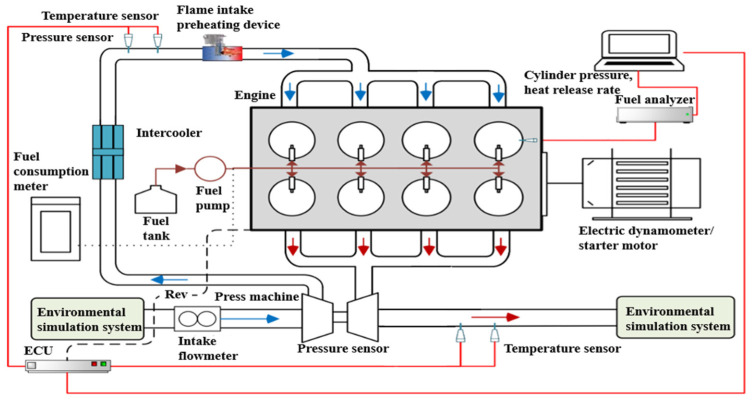
Experimental bench system.

The main equipment of the test bench is an 8-cylinder V-type diesel engine with a firing sequence of 1-8-4-5-7-3-6-2. Acceleration sensors are used to record the vibration acceleration. Figure 5 shows a simplified schematic diagram of the test bench. The experimental steps are as follows: Firstly, in the cold state of the unit, that is, when it has not been started, disassemble the intake and exhaust pipes, cylinder head, fuel injection pipe, return oil pipe, and fault setting cylinder head in sequence. Then loosen the adjusting bolt, quantitatively set it at the intake and exhaust valve gap position of the A4 cylinder with a feeler gauge, and then tighten the adjusting bolt. In this study, the intake and exhaust valve clearances were artificially increased. According to the maintenance manual and engineering practice of this type of diesel engine, the normal valve clearance range (cold state) is 0.25 ± 0.02 mm. When the clearance exceeds 0.27 mm, it is considered abnormal and may lead to performance degradation. In this study, we defined 0.25 mm as the normal baseline, and artificially increased the clearance to 0.30 mm to simulate a typical abnormal condition caused by valve wear or improper adjustment.

At a speed of 1500 rpm under 100% load, performance tests were conducted at 0.25 mm (normal) and 0.3 mm (abnormal), respectively. The vibration signals of the diesel engine’s cylinder head were obtained. In addition, the experiment was implemented in Python 3.10 with PyTorch 2.9.1 and PyTorch Geometric 2.7.0, using a computer with an AMD Ryzen 9 3900X CPU, an NVIDIA GeForce RTX 3060 GPU, and 8 GB of operating memory.

### 4.2. Data Description

The total number of samples in the dataset is 3000, with 1000 samples for each health condition. The original vibration signals were synchronized according to the crank-angle reference and then segmented into cycle-based graph samples. Each graph sample was constructed by extracting five non-overlapping event-centered signal windows corresponding to the key valve-related physical events. Specifically, when both the intake valve clearance and exhaust valve clearance were 0.25 mm, the engine was regarded as being in the normal condition and the corresponding samples were marked as label 0. When the intake valve clearance was 0.30 mm and the exhaust valve clearance was 0.25 mm, the samples represented an intake valve clearance fault and were marked as label 1. When the intake valve clearance was 0.25 mm and the exhaust valve clearance was 0.30 mm, the samples represented an exhaust valve clearance fault and were marked as label 2.

In the initial setup, 50 samples were randomly selected from each category to construct the training set, and 400 samples were randomly selected from each category as the testing set for model evaluation. The specific sample combinations are shown in Table 2. This split was designed to evaluate the diagnostic performance of the proposed method under small-sample training conditions. The relatively large testing set was used to provide a more stable evaluation of the model performance. The training and testing sets were generated after graph-sample construction, and no identical graph sample was included in both subsets. It should be noted that the split in this study was performed at the graph-sample level under the same operating condition, rather than strictly at the independent acquisition-run level. Therefore, although there was no direct duplication between the training and testing samples, cycle samples collected under the same operating condition may still exhibit temporal correlation.

### 4.3. Model HyperParameters Setting

The component graph sample is that the feature length within the node is F = 256, the number of nodes is *n* = 5, and the number of attention heads of the GAT diagnostic model is K = 1. The number of convolutional layers of the graph attention is 2. The specific network layers and structure are shown in Table 3. GATConv and BatchNorm1d are respectively the graph attention convolutional layer and the batch normalization layer, linear is the fully connected layer, and ReLU is the activation function. The hyperparameters during training are shown in Table 4.

### 4.4. Fault Classification Results

**Figure 6:**
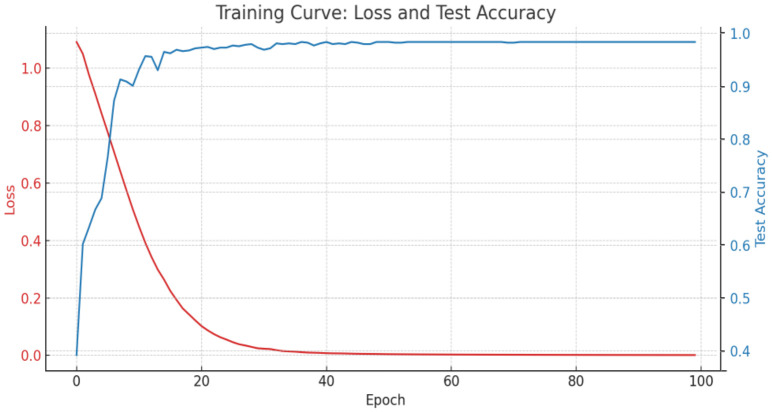
Training loss and test set accuracy curve.

The model was trained under the hyperparameter settings described in Section 4.3. Figure 6 presents the evolution of the training loss and the test accuracy within 100 epochs. During the early stage of optimization, the loss decreases rapidly and then gradually stabilizes. After approximately 20 epochs, the test accuracy stabilizes at around 0.98, indicating that the proposed GAT model can complete high-quality feature learning with good convergence and training efficiency.

**Figure 7:**
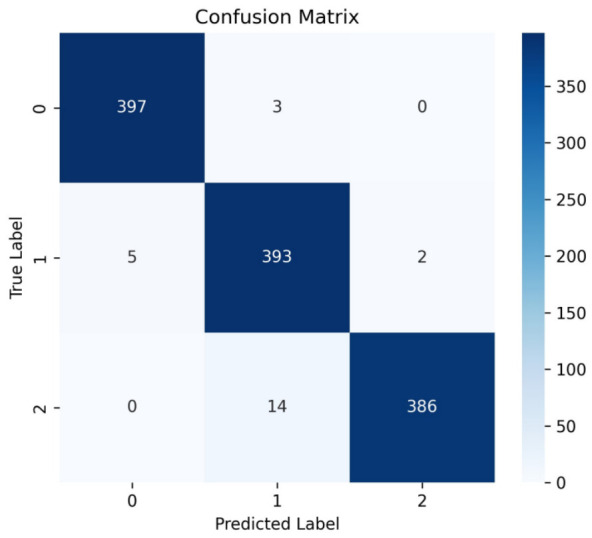
Confusion matrix.

The prediction results are shown in the confusion matrix as illustrated in Figure 7. It can be seen from the figure that the prediction accuracy of the model on each type of sample is relatively high, with an accuracy of over 97%. Among them, category 0 (with normal valve clearance) and category 2 (exhaust valve clearance fault) are classified almost accurately, demonstrating the robustness of the model to edge-class samples. Category 1 (intake valve clearance is relatively large) has a small amount of confusion with adjacent categories, which may be due to the existence of certain ambiguous intervals in the temporal structure characteristics of such samples.

To evaluate the statistical stability of the proposed method, repeated experiments were further conducted using 10 different random seeds. In each run, the random seed controlled the model initialization, sample selection, and mini-batch order. For each health condition, 50 samples were randomly selected for training and 400 samples were randomly selected for testing. Thus, the repeated experiments considered both random initialization and different sample selections.

The repeated experimental results are shown in Table 5. The proposed method achieved a mean best test accuracy of 97.45% ± 0.98% and a mean best macro-F1 score of 97.44% ± 0.98% over 10 runs. The 95% confidence intervals were ±0.61% for both accuracy and macro-F1. The average convergence epoch was 10.2, where convergence was defined as the first epoch at which the test accuracy reached 95% of the best test accuracy in the corresponding run. These results indicate that the proposed method maintains stable diagnostic performance under different random initializations and sample selections.

Considering that diesel engines often face a diagnostic environment with scarce data in actual operation, this paper further tests the classification performance of the GAT model under different training sample sizes. The specific experimental settings are as follows: On the basis of ensuring the balance of the three types of data, five training set scales with the number of samples of each type being 60, 50, 40, 30 and 20 are constructed respectively, and the same test set is used for model evaluation.

**Figure 8:**
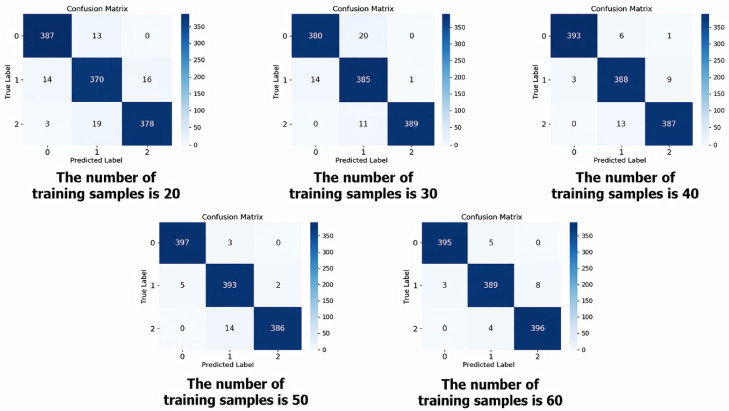
Confusion matrices with different numbers of training samples.

The experimental results are shown in Table 6 and Figure 8. As the number of samples gradually decreases, although the accuracy of the model fluctuates slightly, the overall downward trend is slow and still remains at a relatively high level. Among them, when trained with only 20 samples per category (a total of 60 samples), the model still achieved a test accuracy of 94.58%, demonstrating good small-sample learning capability.

Overall, the GAT model maintains a high recognition accuracy while taking into account the fine discrimination ability among the three types of states, and is suitable for the health status discrimination of multi-state complex devices.

## 5. Discussion

### 5.1. The Influence of Graph Topologies on Model Performance

To deeply analyze the influence of the knowledge embedding graph construction strategy proposed in this paper on the diagnostic performance of graph neural networks, ablation experiments were conducted around two dimensions: the setting of the number of nodes and the construction strategy of node connection relationships, in order to verify the contribution of prior knowledge to the convergence speed and final performance of the model. Firstly, ablation experiments were conducted on the node connection mode based on whether the mechanism knowledge was embedded, namely GAT_5nodes_ordered: An ordered topology diagram was constructed based on the sequence of physical events during the intake and exhaust processes of the engine (intake valve opening/closing, ignition, exhaust valve opening/closing), and clear mechanism prior knowledge was embedded; GAT_5nodes_random: An unordered graph is constructed using a random connection method, ignoring the constraints of the physical structure and relying only on data-driven methods. To ensure a fair comparison, the random graph used the same number of nodes and the same number of directed edges as the proposed graph. The self-loops of all nodes were preserved, and the remaining directed edges were randomly rearranged among different node pairs. Then, an ablation experiment was conducted on the number of nodes based on whether the expression ability of the key structure was retained, GAT_5nodes_ordered (Minimum Key node). Each node corresponds to a typical valve event window; GAT_10 nodes and GAT_15 nodes: Use higher-density sliding window segmentation to build graphs and simulate the graph building strategy in the case of over-segmentation ignoring event knowledge.

**Figure 9:**
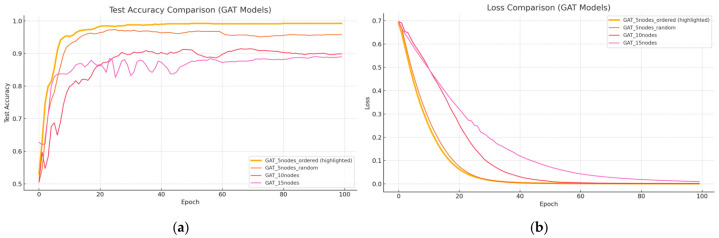
(**a**) Accuracy curves of different graph construction strategies; (**b**) Loss curves of different graph construction strategies.

Figure 9 compares the influence of different graph construction strategies on model convergence and diagnostic performance. As shown in the accuracy and loss curves, the event-driven ordered graph rapidly converges to an accuracy of approximately 98% within the first 10 epochs and maintains stable performance during the subsequent training process. In contrast, the non-prior or randomly connected graph exhibits slower convergence, slightly lower final accuracy, and larger oscillations. The over-segmented graph structures also show less stable convergence than the five-node ordered graph. These results indicate that the performance improvement is not only related to the GAT backbone but is also strongly associated with the prior-knowledge-guided graph topology.

The main reason is that the event-driven graph explicitly constrains information propagation according to the physical occurrence sequence of the working cycle. By treating key valve-related event segments as graph nodes, the model can focus on physically meaningful vibration regions rather than relying on purely data-driven or redundant similarity relationships. Meanwhile, the five-node ordered graph achieves the best convergence speed and accuracy, indicating that event-based node division can compress the input dimension while retaining the key fault-related feature regions. Therefore, the ordered event graph provides a more suitable inductive bias for small-sample valve-clearance fault diagnosis and improves both training efficiency and robustness.

### 5.2. Comparison with Other Fault Diagnosis Methods

In order to comprehensively evaluate the effectiveness of the GAT method guided by prior knowledge, two widely used deep learning models for time series signal processing—CNN and LSTM—were selected as the benchmark models. To ensure a fair comparison, all models used the same training/testing split, optimizer, loss function, learning rate, and number of training epochs. For the CNN and LSTM baselines, key architecture-related hyperparameters, including the number of convolutional filters, convolution kernel size, hidden units, and dropout rate, were tuned within a small candidate range on the validation set. The optimized baseline configurations were then used for the final comparison.

The CNN baseline consisted of two one-dimensional convolutional blocks and one fully connected classification layer. The first convolutional layer contained 32 filters with a kernel size of 5, and the second convolutional layer contained 64 filters with a kernel size of 3. Each convolutional layer was followed by batch normalization, ReLU activation, and max-pooling with a pool size of two. A dropout layer with a dropout rate of 0.5 was used before the fully connected classification layer to reduce overfitting. The LSTM baseline consisted of two LSTM layers with 128 hidden units. The output of the last LSTM time step was passed through a dropout layer with a dropout rate of 0.5 and then fed into a fully connected layer for three-class classification. For all models, Adam was used as the optimizer, cross-entropy was used as the loss function, the learning rate was set to 1 × 10^−4^, the batch size was 32, and the number of training epochs was 100.

**Figure 10:**
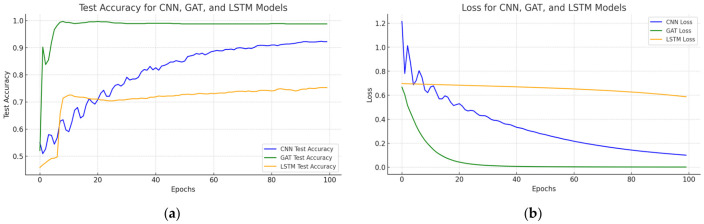
(**a**) Test accuracy curves of the prior-knowledge-guided GAT, CNN, and LSTM; (**b**) Loss curves of the prior-knowledge-guided GAT, CNN, and LSTM.

Figure 10 compares the convergence behavior of the prior-knowledge-guided GAT, CNN, and LSTM. The prior-knowledge-guided GAT reaches an accuracy of approximately 98% within the first 10 epochs and maintains relatively stable performance during subsequent training. In contrast, the CNN baseline shows a slower upward trend and reaches approximately 92% at the 100th epoch, while the LSTM baseline stabilizes at approximately 76%. The loss curves show a similar trend: the prior-knowledge-guided GAT decreases rapidly within the first 10 epochs, whereas CNN and LSTM require more epochs to converge.

In addition to diagnostic accuracy, training efficiency is also an important factor for practical deployment. Although the proposed prior-knowledge-guided GAT introduces graph attention operations, its computational burden is limited because each graph sample contains only five event nodes and a small number of directed edges. For a GAT layer, the computational cost mainly comes from node feature transformation and attention calculation on graph edges. Since the number of nodes and edges in the proposed event-driven graph is small and fixed, the graph computation remains lightweight compared with large-scale graph learning tasks. Compared with CNN and LSTM, the proposed method shows faster convergence because the event-driven graph topology provides a stronger inductive bias for small-sample diagnosis. CNN needs more epochs to gradually learn discriminative local patterns from Euclidean signal sequences, while LSTM requires sequential hidden-state updates and therefore has higher recurrent computation overhead. In contrast, the proposed GAT directly aggregates information among physically meaningful event nodes. Therefore, although the GAT layer introduces attention calculation, the overall training efficiency remains competitive because the model converges within fewer epochs.

These results indicate that representing valve-related vibration segments as graph-structured event data provides a more suitable inductive bias for small-sample valve-clearance fault diagnosis than conventional Euclidean sequence models. More importantly, combined with the ablation results in Section 5.1, the performance improvement is mainly attributed to the prior-knowledge-guided graph construction strategy. When the same GAT backbone was used with a graph structure that did not incorporate mechanism knowledge, its performance was close to that of the CNN baseline.

### 5.3. Interpretability Analysis: Visualization of Attention Weights

**Figure 11:**
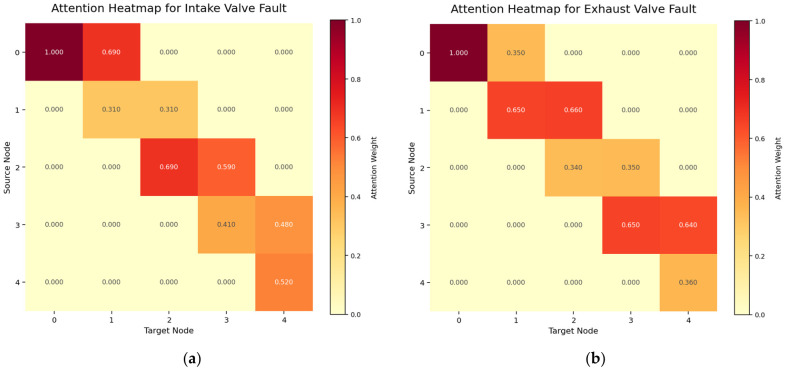
Attention heatmaps under different fault conditions: (**a**) intake valve fault; (**b**) exhaust valve fault. (Node 0: Ignition, Node 1: Exhaust Valve Open, Node 2: Intake Valve Open, Node 3: Exhaust Valve Close, Node 4: Intake Valve Close).

To further analyze the interpretability of the prior-knowledge-guided GAT model, the learned attention weights from the first GAT layer are visualized for different valve-clearance fault conditions. Figure 11a shows the attention heatmap for a representative intake valve fault sample, and Figure 11b shows the attention heatmap for a representative exhaust valve fault sample. Rows denote source nodes and columns denote target nodes. Since the graph nodes are defined according to key physical events in the working cycle, the learned attention weights can be interpreted as the relative importance of event-to-event information aggregation during fault diagnosis.

As shown in Figure 11a, under the intake valve fault condition, relatively higher attention weights are observed around the event transitions associated with the intake-valve-related process. This indicates that the model assigns greater importance to the vibration segments and event relationships that are more sensitive to intake valve clearance variation. In contrast, Figure 11b shows a different attention distribution under the exhaust valve fault condition. The attention weights are more concentrated around the exhaust-valve-related event transitions and their neighboring nodes, suggesting that the model captures vibration response changes caused by exhaust valve clearance variation.

The comparison between the two fault conditions demonstrates that the attention distribution is not fixed, but changes with the health state of the engine. More importantly, the regions with higher attention correspond to physically meaningful event relationships in the valve working process. Therefore, the visualization results provide supporting evidence that the proposed event-driven graph topology improves the interpretability of the diagnostic model: the model not only performs classification, but also highlights the event relationships that contribute more strongly to different valve-clearance faults.

## 6. Conclusions

This paper proposes a prior-knowledge-guided Graph Attention Network method for diesel-engine valve-clearance fault diagnosis. The proposed method starts from the working mechanism of the diesel-engine intake and exhaust system and constructs an event-driven graph representation from cylinder-head vibration signals. Specifically, the vibration signal is synchronized in the crank-angle domain, and key event-centered vibration segments are extracted as graph nodes. Directed edges are then constructed according to the physical occurrence sequence of valve-related events within one working cycle, so that the graph topology explicitly reflects the event evolution process of the valve train.

Based on the constructed event-driven graph samples, a GAT-based diagnostic model is developed to learn graph-level fault representations and classify three valve-clearance states: normal clearance, intake valve clearance fault, and exhaust valve clearance fault. The experimental results under full-load test-bench conditions show that the proposed method achieves high diagnostic accuracy and fast convergence under small-sample conditions. The comparison with CNN and LSTM baselines demonstrates the benefit of introducing graph-structured event representations, while the ablation experiments further indicate that the performance improvement mainly comes from the incorporation of mechanism knowledge into graph construction. The attention-weight visualization also provides an interpretable basis for analyzing event-to-event relationships related to valve-clearance faults.

It should be noted that the experimental validation in this study was conducted under a controlled test-bench condition, with a fixed rotational speed, fixed load, fixed sensor installation position, and a single experimental platform. This setting helps reduce the interference caused by operating-condition variations and allows the proposed method to focus on discriminating valve-clearance-related vibration characteristics. However, it also limits the demonstrated generalization capability of the model. Therefore, the reported results should be interpreted as within-condition diagnostic performance rather than evidence of full robustness under varying industrial operating conditions.

In future work, split-by-run validation, leave-one-recording-out validation, variable-speed and variable-load tests, different sensor positions, and cross-condition transfer validation will be further investigated to more comprehensively evaluate the generalization capability of the proposed method.

## Figures and Tables

**Table 1:** Action phase table of A1 cylinder.

Cylinder	Top Dead Center/Deg	Exhaust Valve Open/Deg	Intake Valve Open/Deg	Exhaust Valve Close/Deg	Intake Valve Close/Deg	Spark Plug Ignition/ Deg
A1	0	122	326	386	580	713

**Table 2:** Sample composition used in the initial experiment.

Name	Value
Complete dataset size	3000
Number of training samples	150
Number of testing samples	1200
Number of training samples for each type	50
Number of testing samples for each type	400

**Table 3:** GAT structure and parameters.

Layer Name	Input Shape	Output Shape
GATConv	[5, 256]	[5, 512]
BatchNorm1d	[5, 512]	[5, 512]
GATConv	[5, 512]	[5, 512]
BatchNorm1d	[5, 512]	[5, 512]
Readout	[5, 512]	[1, 512]
Linear + ReLU	[1, 512]	[1, 256]
Dropout	[1, 256]	[1, 256]
Linear	[1, 256]	[1, 3]
Softmax	[1, 3]	[1, 3]

**Table 4:** Hyperparameters in GAT.

Parameter	Configuration
Batch size	32
Train epoch	100
Learning rate	1 × 10^−4^
Optimizer	Adam
Loss function	Cross-entropy

**Table 5:** Repeated experimental results under different random seeds.

Experiment	Accuracy	F1	Loss	Convergence Epoch
1	0.9750	0.9749	0.0716	9
2	0.9650	0.9649	0.0987	7
3	0.9783	0.9783	0.0587	11
4	0.9850	0.9850	0.0515	11
5	0.9700	0.9699	0.0742	15
6	0.9917	0.9917	0.0360	9
7	0.9667	0.9666	0.1237	10
8	0.9817	0.9817	0.0621	9
9	0.9600	0.9598	0.1085	7
10	0.9717	0.9716	0.0721	14
Average	0.9745	0.9744	0.0757	10.2

**Table 6:** The test accuracy of the GAT model under different small sample sizes.

Samples Per Class	Total Number of Training Samples	Test Accuracy
60	180	0.9833
50	150	0.9800
40	120	0.9733
30	90	0.9617
20	60	0.9458

## Data Availability

Data are available from the corresponding author upon reasonable request.
